# The dental anomaly: how and why dental caries and periodontitis are phenomenologically atypical

**DOI:** 10.1186/s13010-019-0084-5

**Published:** 2019-10-26

**Authors:** Dylan Rakhra

**Affiliations:** Department of Philosophy, University of Bristol, Bristol, United Kingdom

**Keywords:** Dentistry, Caries, Periodontitis, Disease, Illness, Naturalist, Normativist, Phenomenology, Philosophy, Prevention

## Abstract

**Background:**

Despite their shared origins, medicine and dentistry are not always two sides of the same coin. There is a long history in medical philosophy of defining disease and various medical models have come into existence. Hitherto, little philosophical and phenomenological work has been done considering dental caries and periodontitis as examples of disease and illness.

**Methods:**

A philosophical methodology is employed to explore how we might define dental caries and periodontitis using classical medical models of disease – the naturalistic and normativist. We identify shared threads and highlight how the features of these highly prevalent dental diseases prevent them fitting in either definition. The article describes phenomenology and the current thought around the phenomenology of illness, exploring how and why these dental illnesses might integrate into a phenomenological model.

**Results:**

We discover that there are some features particular to dental caries and periodontitis: ubiquity, preventability and hyper-monitorablility. Understanding the differences that these dental diseases have compared to many other classically studied diseases leads us to ethical questions concerning how we might manage those who have symptoms and seek treatment. As dental caries and periodontitis are common, preventable and hyper-monitorable, it is suggested that these features affect the phenomenology of these illnesses. For example, if we experience dental illness when we have consciously made decisions that have led to it, do we experience them differently to those rarer illnesses that we cannot expect? Other diseases share these features are discussed.

**Conclusions:**

This paper highlights the central differences between the classical philosophical notion of disease in medicine and the dental examples of caries and periodontitis. It suggests that a philosophical method of conceptualising *medical* illness - phenomenology - should not be applied to these dental illnesses without thought. A phenomenological analysis of any dental illness is yet to be done and this paper highlights why a separate strand of phenomenology should be explored, instead of employing those that are extant. The article concludes with suggestions for further research into the nascent field of the phenomenology of dental illness and aims to act as a springboard to expose the dental sphere to this philosophical method of analysis.

## Introduction

Our definitions of disease and illness are constantly in flux. From Epicurus to Cooper, scientists and philosophers have argued about how disease and illness can be conceptualised [1, 2]. In the clinical dental sphere, the discourse surrounding dental disease is, for the most part, naturalistic and pathophysiological - with a selection of practitioners favouring a microbiological stance [3–5]. This method of understanding disease aligns with the first microbiological theories presented in dentistry and the work of naturalist philosopher Christopher Boorse, who considers health and disease to be entirely biological, *value-free* notions – his Biostatistical Theory (BST) [6, 7]. Normativists, such as Cooper [2], posit that disease is a sociocultural phenomenon and is *value-laden*. A plethora of other accounts – hybrid theories - have sprung into existence that span the two camps. Despite this, there are various threads connect these extant models.

In considering illness, phenomenology – a branch of philosophy concerned with human lived experience – has been helpful in providing a philosophical framework for understanding its experience. Phenomenologists such as Husserl and Merleau-Ponty have explored in detail how people experience their world and body; Carel, Toombs and Leder, amongst others, have explored how illness affects this lived experience [8–11]. Many areas of medicine have ongoing phenomenological debates where the experience of illness is discussed - for example, old age, suffering, mental illness [12] - however, whilst the literature on phenomenology of illness is growing, there exists a lacuna: the phenomenology of dental illness.

Whilst much work has been done to understand the anatomical, physiological, pathological and socioeconomical features of medical and dental disease and illness, little attention has been given to how the *nature* of the two might differ. Central to this article are dental caries and periodontitis, the most commonly managed diseases of the primary care dentist. Dental caries, is the dental term referring to tooth decay in any of its forms. Periodontitis or, colloquially, gum disease, is the chronic inflammation of the gums causing destruction of the bony support of the teeth resulting in tooth movement and loss. The intuitive and easy trap to fall into, as also recognised by Holden and others [13], is that medicine and dentistry have shared origins and, as such, their subject matter can be subject to the same analyses.

The first section of this article will outline, and give examples of, the principal notions of naturalist and normativist definitions of disease and draw out connecting threads between them. Section two will reveal how dental caries and periodontitis do not comfortably fit into any camp, given the central properties that separate them from many other medical diseases as separate to the above dental diseases. The end of the section considers other diseases that the present analysis may apply to and how advancements in technology and medical techniques might widen this group of diseases. The final section provides an outline of phenomenology and the phenomenology of illness. It discusses the capacity for the phenomenological exploration of these dental illnesses as distinct from general medical illness. The article concludes with suggestions for further work using existing phenomenological frameworks in an attempt to serve as a conduit into the nascent field of the phenomenology of dentistry.

## Naturalism and Normativism

Boorse claims that a disease is an individual’s altered internal state leading to an ‘impairment of normal functional ability [ … ] below a typical efficiency’: where normal function is based on what is statistically typical for the individual within a reference class [7]. A Boorsian reference class is defined as a ‘class of organisms of uniform functional design; specifically an age group or a sex of a species’ [7]. In essence, irrespective of how I view myself, if I am not functioning as well as the other humans in my peer group, I am diseased.

One of the many challenges to this view is how widespread diseases fit in. A common counterexample is that of dental caries [2, 14]. Boorse *himself* notes this as being a counterexample. However, he explains that, given that so few examples of these highly prevalent diseases are identified (tooth decay and lung irritation), we should not ‘bifurcate’ the definition [15]. As such, he goes on to explain that tooth decay *is* normal [15]. Within the wide reference class of the United Kingdom, just over a third of adults – still a minority - had obvious dental decay in the most recent Adult Dental Health Survey (ADHS) [16]. It is unclear how a minority of people with a highly prevalent pathology can be considered to be normal as well as the other two-thirds of people without tooth decay present. Whilst it is not the purpose of this section to provide a review of all the criticisms levelled at Boorse’s conceptualisation of disease, the above example, dozens of other criticisms and two rebuttals spanning more than 35 years suggest that the Boorsian BST is not watertight [15, 17].

Normativists challenge a biostatistical conception of disease and dispense with value-free judgements. Cooper posited that disease is a normative phenomenon and must be conceptualised with strong sociocultural considerations [2]. Despite this significant difference, both the naturalist and the normativist notions of disease have parallels. They share a similar idea of reference classes. King [18] presents his ideas of ‘statistical norms’, Cooper highlights that a diseased individual is defined as being ‘roughly worse off than the majority of humans of the same sex and age’ [2] – with this, she introduces the idea of the afflicted person being *unlucky*. She continues to explain that the disease must be a bad thing to have and have potential medical treatment available [2]. Under the normativist characterisation, we have the ability to make value judgements on our own pathologies when considering if an individual in our community is diseased – society, not biology, is judge and jury.

Theories that aim to combine the above have appeared [19, 20]. Ereshefsky controversially suggested we should do away with the terms ‘health’ and ‘disease’ in technical discussion [19]. It is not within the scope of this article to expand on all extant accounts of health, illness and disease, however, what is important is that most of them share a recognition of reference classes and the relative rarity of disease with, some recognising the undesirability of disease.

That disease is relatively rare and undesirable seems intuitive given that diseases and illnesses are not synchronically thought to be something that all people have, expect to have, or are content with having. Almost all of us will eventually die of some disease and, diachronically speaking, should expect to become afflicted with disease as we enter old age.^1^ However, apart from this, we anticipate and desire to live in good health.^2^

## The Dental Anomaly

We have seen how, even within two extremely distinct accounts of disease, disease is some pathology that is a variation from the majority that may cause some harm to life and, in some accounts, is considered problematic to have. It follows that if disease befalls the minority and causes disruption, I do not expect to become diseased today - although I would expect to encounter disease when considering my life as a whole.

Dental caries and periodontitis occupy an interesting niche here; they are near ubiquitous in some form. In the United Kingdom, 90% of adults have some manifestation of the above [16]. It is also almost entirely preventable and hyper-monitorable – that is to say, there are sensitive, quick, simple and non-invasive tests to find these pathologies, even in their early stages.^3^ If a set of diseases are near ubiquitous, they cannot be said to be rare; if they are preventable and hyper-monitorable, they cannot be said to be unexpected. If dental caries and periodontitis do not share the basic tenets of many other medical diseases, how useful can applying these notions be when considering the dental discourse?

Boorse, using his BST, would explain the above by saying that these dental diseases are simply not diseases. He may point out that the reference classes shift such that only severe dental decay causing unbearable pain or advanced periodontitis causing tooth movement and loss are diseases. This seems counterintuitive: if I was diagnosed with mild periodontitis and tooth decay, even if it were subclinical, I would certainly consider myself to have a disease and accept treatment or change my lifestyle and habits accordingly. Indeed, if the condition was dismissed as health and no amends made, the progressive nature of caries would mean that the severe symptoms would soon enough present. That we know what constitutes a disease and if, when or how it should be treated is extremely important for the ethics of any medical profession, therefore, it is not pragmatic to simply re-label those with dental pathology as ‘not diseased’ when many of the same people may want to seek treatment to remove or ameliorate their pathology.

It might be the case that dental illness is best taken normativistically. Would, then, my above intuition that I am diseased and my concern for my subclinical dental pathologies be justified and, thus, classified as a disease? On the surface, it would seem so. I’d certainly consider my lot to be bad and would seek potential medical treatment but it is not entirely clear I could consider myself *unlucky*. Preventability and monitorability are crucial here.

### Prevention and monitoring of dental disease

Cooper explains that we can only ‘consider someone to be diseased if they could reasonably have hoped to have been otherwise’ [2]. If a group of diseases are preventable, for the most part, it is lifestyle factors that lead to them. It is ultimately my decision to eat a diet high in sugars and ultimately my decision to conduct a poor oral hygiene regime. Given the above, it cannot be the case that it is unlucky for me to experience a disease that these high-risk behaviours lead to. Cooper clarifies that her definition of unluckiness is as being ‘judged by the uninformed layman’ and not medical science but there must be an extent to which this layman is uninformed. The notion of taking care of one’s dental health is not esoteric – it is widespread knowledge that maintaining good oral hygiene and a low sugar diet and so on lead to good oral health. Thus, one cannot be reasonably considered unlucky to develop dental caries or periodontitis.^4^

An exception must be made of those with concomitant or congenital disease that *prevent* prevention, for example, Parkinsonian patients with dyskinesia – the impairment of voluntary movement. This cohort classically find it difficult or impossible to carry out the highly dextrous task of brushing their teeth. In this case, I put forward that a primary disease that falls into the normal medical conceptualisation of disease applies here and the preventable dental disease becomes secondary, a sequela of the primary.

It could be argued that we do not know the status of our bodies at all times and, as such, how can we guide the prevention of disease? I might live my life exercising each day and eating healthily in the attempt to stave off angina and heart attack but I cannot ask my general practitioner (GP) to continuously measure the levels of atherosclerotic plaques in my arteries.^5^ If we followed the guidance of every public health campaign, we might try to engage in a perfectly abstemious life, purely in the hope that no disease befalls us but this is impractical and a realistic balance must be achieved. This is where the monitorability of caries and periodontitis become important. Modern dentistry has the capacity to make a direct assessment of the presence of these dental pathologies in any area of the mouth in a matter of minutes and in a non-invasive manner. For example, a dentist can probe the gums with specialised yet simple tools and assess presence of periodontitis with no ill-effect to the patient. It is relatively easy to prevent and know you are effectively preventing dental disease.

In summary, if I act in a way that is detrimental to my oral health and make no attempt to discover any of the dental pathologies described – or, indeed, choose to ignore the diagnoses when they are made - I cannot be considered to be unlucky to have these diseases. It follows, then, that normativist conceptions of the dental diseases in question do not fit. In combination with how the ubiquity of these dental pathologies prevent a naturalistic definition being used for dental disease – how can we have meaningful philosophical discussion of these dental diseases?

### Lifestyle disease and the future

Though this article puts dental caries and periodontitis in the spotlight, it is unclear that there is anything inherent to these pathologies themselves that results in them being the only ubiquitous, monitorable and preventable diseases to which this analysis might apply. Examples such as Type 2 Diabetes Mellitus (T2DM), smoking-related diseases and some sexually-transmitted diseases are also eminently preventable with various lifestyle changes. Dermatological diseases such as acne or eczema are equally, if not more, monitorable than dental caries and indeed various forms of allergy are extremely common. There may also be diseases that share all of these characteristics.

We must also consider here the influence of future technologies and discoveries on body monitoring and disease prevention. For example, there is no inherent characteristic of dental caries that makes it monitorable. It is simply that the ease of non-invasive access and current scientific knowledge and technology allow it to be. Advancements in the future might provide equally simple direct monitoring of other preventable pathology. The example used above may apply: the monitoring of atherosclerotic plaques in my arteries. If this could be cheaply and efficiently quantified, tailored advice on lifestyle change and personalised treatments might be easily given. In this case, the current analysis being expounded for dental caries and periodontitis may also apply to those diseases.^6^ It is not within the scope of the article to delve deeper into each of these examples but there is further work to be done considering if and how other diseases and pathologies share these three central characteristics.

## Phenomenology of Dental Illness

We have seen how dental caries and periodontitis do not fit definitions of classical medical disease. If we find it difficult to settle on what is and is not a disease, pragmatic, financial and ethical questions arise around how we might diagnose, treat and manage those who have symptoms and seek treatment. There are clear moral and ethical quandaries here; are we under-treating or over-medicalising? How can we navigate this issue?

Although phenomenology is not a method of defining disease, phenomenologists of illness argue that even with sound definitions of disease (whether naturalist, normativistic or hybrid), it is not sufficient to understand the illness experience. In a situation where these definitions fall short – as presented above with caries and periodontitis – we might look to other methods of understanding this experience. As such, I suggest a phenomenological analysis of these dental diseases, considering them as dental illnesses*,* specifically dental *illnesses-as-lived*, would allow more meaningful discussion of these dental pathologies and help us answer the ethical and moral questions outlined above. As of yet, application of phenomenological analysis to dental illness is extremely sparse, if not entirely absent.

Phenomenology is a method of philosophical analysis concerned with the structure and content of human lived experience instead of asking of what truly exists or, more generally, the metaphysical constituents of nature. In essence, it is because *we* experience things that they are endowed with meaning and significance *qua phenomena*. Phenomena appear to a consciousness; meaning is not an inherent property of an object that is naturally there for us to encounter [22]. Phenomenology can describe any experience, for example, the experience of listening to a melody or observing a cat’s movements [23]. When applied to disease, it emphasises how disease is *experienced*, i.e. as illness – how disease appears to us, and blurs the focus on the inquiry into biological dysfunction.

As always, it should be noted that the empirical study of disease is, of course, important and has been extremely successful in the advancement of medical knowledge and responsible for curing many diseases and saving many lives. The use of phenomenology to study illness is not designed to replace empirical scientific investigation into disease and medicine; one must be mindful of the distinction between medical science and clinical medicine. Though the naturalistic definition of disease has been shown to poorly describe dental diseases such as caries and periodontitis, epistemological naturalism and dental science are to be augmented by research into the phenomenology of dental illness and not contested by it. The use of phenomenology in this debate has been criticised more recently with the crux of the argument showing that simply a wider take on naturalism provides a holistic and patient-centred view of disease [24–26]. However, the extant views on what constitutes diseases – from naturalistic to normativistic - are fundamentally not sufficient for use with the abovementioned dental diseases, as outlined above. As such, phenomenology may help us better understand it.

Discussion of illness, in leiu of disease, takes into account not only the pathologies – but the entire gamut of history and context within which it is experienced. This leads to richer descriptions of the experience of illness that encompass society, culture and what the experience of a disease *means* to a person. Through a phenomenological lens, toothache from dental decay is *not* simply late-stage bacterial advancement through tooth tissue towards a set of nerve endings causing pain but also a disruption to eating, to socialising, a jarring reminder of childhood experiences, anxieties and the length of a sleepless night, an extra item in the to-do list, an extra journey after work, a potentially larger hole in the wallet and impending judgement from friends or family or, even, from oneself.

Few others have loosely alluded to highlighting the experiencer in dental illness. Hofman and Eriksen, in an exploration of ethics in dentistry, introduce a concept of *sickness* to form a triad of disease, illness and sickness [27]. Here, various perspectives of the pathology are considered – that of the physician, society and, importantly, the patient themselves – in deciding on whether or not to treat. They do not discuss phenomenology per se but acknowledge that the ‘illness’ part of the triad has ‘ethical primacy’; it is the patient’s experience that is the most important factor in dental ethics [27]. This is echoed in more recent explorations into the phenomenology of illness:

‘[ … ] illness is the most important element of the disease-illness coupling. We care about *physiological* dysfunction primarily when it causes us *pain or discomfort*, or prevents us from doing certain things’ [8]. [emphasis added]

Phenomenology’s use in providing a framework for philosophically understanding illness has gained great traction but it is not entirely clear that we can simply use the same phenomenological research in the discussion of those who have diagnoses of dental caries and periodontitis, given that they are so different and poorly described by classical medical definitions of disease. For example, dental caries is common, preventable and monitorable; the great majority of us know how to avoid it and why it happens. This knowledge *itself* may change the experience of the illness. If we experience dental illness when we have consciously made avoidable decisions that have led to it, do we experience them similarly to those rarer illnesses that we cannot expect, e.g. a diagnosis of pancreatic cancer? It might even be the case that precisely *because* dental caries is so preventable, its illness experience may initially be lined with notions of failure and lack of self-discipline. It would be exceedingly counterintuitive to suggest one might have a similar self-judgement of failure-to-prevent when diagnosed with a genetic condition such as Huntington’s. Does prevention of illness, as a whole, warrent its own phenomenological analysis? As alluded to in the previous section, these differences in the illness experience may be shared with other illnesses that have similar central characteristics, for example T2DM, but through phenomenology, any similarities or differences that are uncovered within a spectrum of illnesses may even *inform* our discussion of disease and its management instead of function to belittle the pathology as normal or not worthy of treatment.

Carel has shown how having a thorough phenomenological understanding of illness can provide us with insight into how we interact, manage, and treat disease and, indeed, human experience [8, 28]. This approach leads to more holistic medicine and, in the case of dental caries and periodontitis, alleviates the pressure for a firm definition of these diseases. If we can apply phenomenology to these dental illnesses, we may be able to further expand the usefulness of phenomenology in wider illness. Further work to assess how dental illness might align with or deviate from current phenomenological frameworks must be done before we can comfortably begin exploring the phenomenology of these dental illnesses.

## Conclusion

Despite having similar past origins and contemporary methods of practice, it is not sensible to assume that the disciplines of medicine and dentistry are similar enough such that an analysis into the subject matter of one is interchangeable with the other. This article first outlined how dental caries and periodontitis differ in fundamental ways to many other medical diseases, by expounding the most popular schools of thought in defining disease – the naturalist and normativist – and showing that they poorly encompass these dental diseases. By being almost ubiquitous, preventable and hyper-monitorable, it aligns poorly with the concept of deviation from a reference class and with notions of being unlucky. Discussion of how dental caries and periodontitis might only be examples of a gamut of diseases that share these similar characteristics followed, highlighting T2DM, smoking-related diseases, some sexually-transmitted diseases, acne, eczema and various forms of allergy.

How can we meaningfully discuss the treatment and experience of dental caries and periodontitis if it is disputed that they can be classed as diseases? Ethical decisions can only be made about when, why and how to treat various diseases if all can agree that these *are* diseases. The final section of the article outlined the phenomenological method and showed how it can give us another mode of conceptualising disease *as it is lived*; as illness. Though not designed to replace philosophical definitions of disease (such as naturalism or normativism), phenomenology allows us to have a meaningful discourse about dental illness, where we could discuss the *experience* of a illness and the effect it might have on a person’s life, for example, the disruptions and anxieties that it may cause. This, in turn, informs and aids a practitioner to manage patients and their illnesses in an ethical manner.

However, for the same reason that some of the characteristics of these dental diseases preclude them being well described using the classical medical models, these same differences are likely to change – or at least modulate – how dental illness is experienced. Society, medicine, culture, habits and knowledge are intimately linked with how these differing characteristics of dental disease affect dental illness as they appear to consciousness. The example of preventative knowledge and aspects of felt failure was given. It is unclear that the extant phenomenological frameworks of illness will simply apply to dentistry without modification. As such, we concluded with suggestions for further work into how dental illness might interact with current phenomenological frameworks in the hope of starting a debate in which the phenomenology is applied to the dental sphere.

## Data Availability

Not applicable.

## Abbreviations

ADHS: Adult Dental Health Survey
BST: Biostatistical Theory
GP: General Practitioner
T2DM: Type 2 Diabetes Mellitus

## Footnotes

1 With the exception of those who die traumatically, due to accident. See [21] for an exposition of synchrony and diachrony in illness – the former referring to a point in time in one’s life and the latter to the entirety of one’s life.

2 Of course, there will be minor ailments that we come across with relative regularity: a cold or a papercut. These afflictions are undesirable and expected to occur occasionally, however, they are fleeting – with a natural history of between hours or a few days - and commonly leave no signs or symptoms.

3 A note must be made here that there are factors out of one’s control that influence the rate of dental caries and periodontitis. For example, a person with a genetic predisposition to aggressive periodontitis living in a place where access to tools to maintain excellent oral hygiene are not available would not be able to prevent periodontitis. Equally, child with a congenitally low salivation rate who is put to bed with drinks high in sugar may not be able to prevent dental caries. However, it is physically, if not logistically, possible to successfully prevent nearly all of the cases of these pathologies, especially with consistent and close aid by a dentist.

4 One might posit that being born into or living in an environment that cultures these diseases is unlucky and this justifies the normativist’s condition. However, this unluckiness is not a feature of the disease, in the same way one might consider a stroke to be unlucky. The unlucky status is a misfortune that affects one’s life globally and has a cascade of effects – some of which might lead to manifestation of disease. The normativistic ‘unluckiness’ concerns that situation where disease manifests contra sociocultural expectations.

5 Although surrogate markers do exist, e.g. blood pressure, they are not perfect, direct measures of the internal pathologies.

6 I would like to thank an anonymous reviewer of an earlier draft of this article for this point of qualification.
